# Trastuzumab emtansine: mechanisms of action and drug resistance

**DOI:** 10.1186/bcr3621

**Published:** 2014-03-05

**Authors:** Mark Barok, Heikki Joensuu, Jorma Isola

**Affiliations:** 1 Laboratory of Molecular Oncology, University of Helsinki, Biomedicum, Haartmaninkatu 8, Helsinki FIN-00290, Finland; 2 Department of Oncology, Helsinki University Central Hospital Haartmaninkatu 4, Helsinki FIN-00029, Finland; 3 MioMediTech, University of Tampere, Biokatu 6, Tampere 33014, Finland

## Abstract

Trastuzumab emtansine (T-DM1) is an antibody-drug conjugate that is effective and generally well tolerated when administered as a single agent to treat advanced breast cancer. Efficacy has now been demonstrated in randomized trials as first line, second line, and later than the second line treatment of advanced breast cancer. T-DM1 is currently being evaluated as adjuvant treatment for early breast cancer. It has several mechanisms of action consisting of the anti-tumor effects of trastuzumab and those of DM1, a cytotoxic anti-microtubule agent released within the target cells upon degradation of the human epidermal growth factor receptor-2 (HER2)-T-DM1 complex in lysosomes. The cytotoxic effect of T-DM1 likely varies depending on the intracellular concentration of DM1 accumulated in cancer cells, high intracellular levels resulting in rapid apoptosis, somewhat lower levels in impaired cellular trafficking and mitotic catastrophe, while the lowest levels lead to poor response to T-DM1. Primary resistance of HER2-positive metastatic breast cancer to T-DM1 appears to be relatively infrequent, but most patients treated with T-DM1 develop acquired drug resistance. The mechanisms of resistance are incompletely understood, but mechanisms limiting the binding of trastuzumab to cancer cells may be involved. The cytotoxic effect of T-DM1 may be impaired by inefficient internalization or enhanced recycling of the HER2-T-DM1 complex in cancer cells, or impaired lysosomal degradation of trastuzumab or intracellular trafficking of HER2. The effect of T-DM1 may also be compromised by multidrug resistance proteins that pump DM1 out of cancer cells. In this review we discuss the mechanism of action of T-DM1 and the key clinical results obtained with it, the combinations of T-DM1 with other cytotoxic agents and anti-HER drugs, and the potential resistance mechanisms and the strategies to overcome resistance to T-DM1.

## Introduction

Overexpression and amplification of human epidermal growth factor receptor-2 (HER2, ErbB2) is present in 15 to 20% of primary human breast cancers [1]. In the past, patients with HER2-positive breast cancer generally had unfavorable outcome [2], but this changed radically after discovery of trastuzumab, a recombinant humanized monoclonal antibody that binds to the extracellular subdomain IV of HER2. Trastuzumab showed substantial anti-tumor efficacy in both preclinical and clinical trials [3,4], and introduction of trastuzumab for the treatment of HER2-positive breast cancer can be considered a milestone in medical oncology [4,5]. However, resistance to trastuzumab eventually emerges in the great majority of patients treated [6].

Several other HER2-targeted agents have been evaluated in clinical trials since the introduction of trastuzumab in 1998. Lapatinib, an orally administered small molecule inhibitor of the HER1 and HER2 tyrosine kinases, was found to be superior in combination with capecitabine compared with capecitabine alone in the treatment of metastatic breast cancer (MBC) that had progressed after trastuzumab-based therapy [7]. As to trastuzumab, resistance to lapatinib develops frequently among patients who initially respond [8]. Recently, pertuzumab, a recombinant humanized monoclonal antibody that binds to subdomain II of the extracellular portion of HER2 and inhibits receptor dimerization, was found to be more effective in combination with trastuzumab and docetaxel compared with placebo, trastuzumab and docetaxel as first-line treatment of HER2-positive MBC [9].

Despite these new therapeutic options, HER2-positive MBC still remains an incurable disease. In this review we discuss the mechanisms of action of trastuzumab emtansine (T-DM1), a novel agent that has challenged in efficacy and safety all existing systemic therapies for HER2-positive MBC, and the resistance mechanisms to it. T-DM1 is an excellent example of a principle suggested already in the 1970s to use antibodies as carriers of drugs to highly specific targets [10].

## Trastuzumab emtansine, a HER2-targeted antibody-drug conjugate

Antibody-drug conjugates (ADCs) are a means to deliver cytotoxic drugs specifically to cancer cells. The delivery is followed by internalization of the ADC and release of free, highly active cytotoxic agents within cancer cells, leading eventually to cell death. The components of an effective ADC typically consist of: (i) a humanized or human monoclonal antibody that selectively and specifically delivers a cytotoxic agent to cancer cells by evoking receptor-mediated endocytosis; (ii) a cytotoxic agent that will kill the cell; and (iii) a linker that binds the cytotoxic agent to the antibody.

The first ADC targeting the HER2 receptor is T-DM1 (ado-trastuzumab emtansine; T-MCC-DM1; Kadcyla®), which is a conjugate of trastuzumab and a cytotoxic moiety (DM1, derivative of maytansine). T-DM1 carries an average of 3.5 DM1 molecules per one molecule of trastuzumab. Each DM1 molecule is conjugated to trastuzumab via a non-reducible thioether linker (*N*-succinimidyl-4-(*N*-maleimidomethyl) cyclohexane-1-carboxylate; SMCC, MCC after conjugation) [11].

### Mechanisms of action of T-DM1

**Figure 1:**
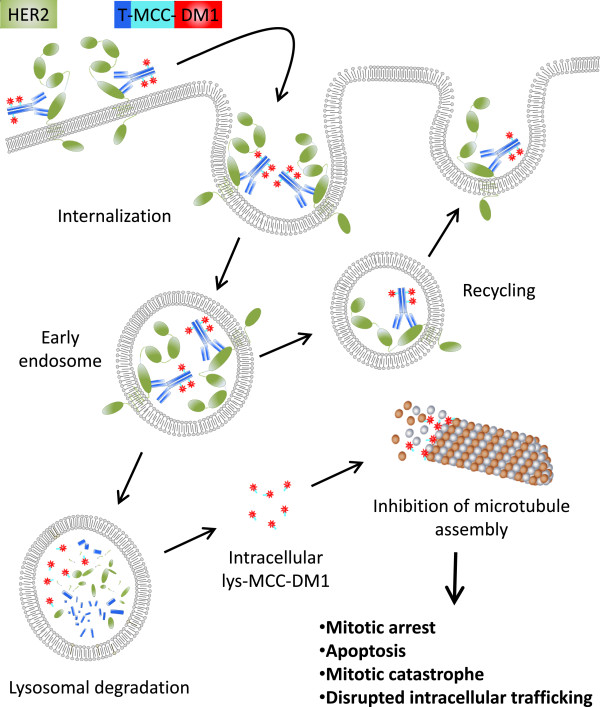
**Intracellular trafficking of trastuzumab emtansine (T-DM1).** Binding of T-DM1 onto human epidermal growth factor receptor-2 (HER2) on the plasma membrane is followed by entry of the HER2-T-DM1 complex into the cell via receptor-mediated endocytosis. Internalized endocytic vesicles form early endosomes. The load of early endosomes can be recycled back to the cell membrane or the early endosome can mature to a lysosome. Release of DM1 occurs as a result of proteolytic degradation of the antibody part of T-DM1 in the lysosomes. Intracellular lysine (lys)-MCC-DM1 inhibits microtubule assembly, causing mitotic arrest, apoptosis, mitotic catastrophe, and disrupted intracellular trafficking. MCC, non-reducible thioether linker.

Binding of T-DM1 to HER2 triggers entry of the HER2-T-DM1 complex into the cell via receptor-mediated endocytosis [12,13]. Since the non-reducible linker is stable in both the circulation and the tumor microenvironment, active DM1 release occurs only as a result of proteolytic degradation of the antibody part of T-DM1 in the lysosome [11,14]. Following release from the lysosome, DM1-containing metabolites inhibit microtubule assembly, eventually causing cell death [15] (Figure 1).

Linkage of DM1 to trastuzumab does not affect the binding affinity of trastuzumab to HER2 [16,17], nor does it reduce the inherent anti-tumor effects of trastuzumab [16,18]. Consequently, T-DM1 has mechanisms of action consisting of the anti-tumor effects related to trastuzumab and those associated with intracellular DM1 metabolites (Table 1).

**Table 1:** Mechanisms of action of trastuzumab and trastuzumab emtansine

	**Mechanism of action**	**Mechanism causing trastuzumab resistance**
**Trastuzumab**		
Fab-mediated	Down-regulation of HER2 on the plasma membrane [19]	Masking of trastuzumab binding epitope of HER2 [20,21]
	Inhibition of HER2 ectodomain shedding [22]	Expression of p95HER2 [23]
	HLA-I-restricted antigen presentation of HER2 [24]	Activation of the IGF-IR pathway [25]
	Inactivation of the PTEN-PI3K/AKT pathway [26]	Defects in the PTEN-PI3K/AKT pathway [26]
	Induction of apoptosis [19]	Overexpression of cyclin E [27]
	Inhibition of angiogenesis [28]	Autocrine production of EGF-related ligands [29]
Fc-mediated	ADCC [30]	Impaired ADCC [31]
**T-DM1**		
Trastuzumab part		
Fab-mediated	Inhibition of HER2 ectodomain shedding [16]	
	Inhibition of PI3K/AKT signaling pathway [16]	
Fc-mediated	ADCC [16,18]	
DM1 part	Mitotic arrest [11]	
	Apoptosis [11,17,18]	
	Mitotic catastrophe [18]	
	Disruption of intracellular trafficking [18]	

ADCC, antibody-dependent cell-mediated cytotoxicity; AKT, protein kinase B; EGF, epidermal growth factor; HER2, human epidermal growth factor receptor-2; HLA, human leukocyte antigen; IGF-IR, insulin-like growth factor-I receptor; PI3K, phosphatidylinositol 3′-kinase; PTEN, phosphatase and tensin homolog; T-DM1, trastuzumab emtansine.

### Trastuzumab-mediated effects

Both trastuzumab and T-DM1 inhibit HER2 receptor signaling, mediate antibody-dependent cell-mediated cytotoxicity, and inhibit shedding of the extracellular domain of HER2 [16,18]. Although the anti-tumor effects of DM1 are more pronounced than those of trastuzumab [16], trastuzumab-mediated effects should not be underestimated and might be particularly important when the target cells do not undergo rapid apoptotic death caused by DM1. This may be common in the clinic, where trastuzumab therapy of MBC often lasts for several months or years, and continuation of trastuzumab therapy beyond breast cancer progression on trastuzumab-containing systemic therapy may still be beneficial [32,33].

### DM1-mediated effects

**Figure 2:**
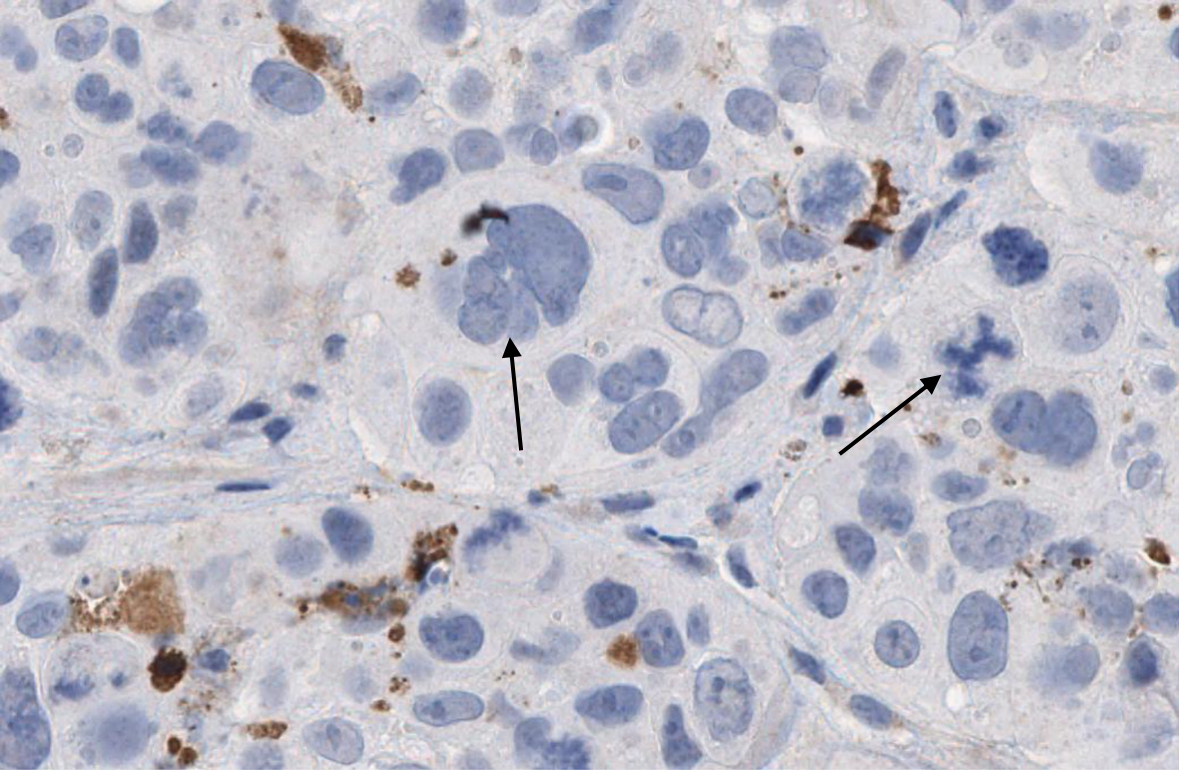
**Histological findings in a human epidermal growth factor receptor-2-positive, trastuzumab and lapatinib-resistant breast cancer (JIMT-1) xenograft following trastuzumab emtansine treatment.** Numerous apoptotic cells are present (stained brown with CytoDeath staining). Hematoxylin counterstain reveals multinucleated giant cells and pathological mitoses (arrows), which are hallmarks of mitotic catastrophe. Mitotic catastrophes were absent in trastuzumab-treated tumors.

At least four molecular mechanisms have been suggested for DM1 anti-tumor activity. First, active DM1 metabolites disrupt the microtubule networks of the target cells, which causes cell cycle arrest at the G_2_-M phase and apoptotic cell death [11,18]. Second, prolonged treatment of breast cancer xenografts with T-DM1 caused both apoptosis and mitotic catastrophe, the latter being identified as presence of cells with aberrant mitotic figures and a giant multinucleated structure (Figure 2) [18]. Third, disruption of microtubule network-mediated intracellular trafficking may occur. Microtubule targeting agents often disrupt intracellular trafficking via microtubules [34,35], and prolonged treatment with T-DM1, but not with trastuzumab, caused defective intracellular trafficking of HER2 in a preclinical breast cancer model [18]. Impaired intracellular trafficking may be an important mechanism of action of T-DM1, particularly in non-dividing cells. Finally, as we discuss below, free intracellular DM1 may lead to cell death in a concentration-dependent manner.

## Activity of T-DM1 in preclinical models and clinical trials

A comprehensive review of the efficacy and safety results obtained with T-DM1 is beyond the scope of the current review but, in brief, T-DM1 has shown substantial anti-tumor efficacy in preclinical studies and clinical trials. T-DM1 has superior activity compared with trastuzumab on trastuzumab-sensitive breast cancer cell cultures and tumor xenografts (Additional file 1) [11,18]. Importantly, T-DM1 is effective in *in vitro* and *in vivo* models of trastuzumab-resistant breast cancer, and in trastuzumab and lapatinib cross-resistant breast cancer models (Additional file 2) [11,18].

A key clinical trial to investigate the efficacy and safety of T-DM1 in the treatment of breast cancer was the EMILIA study, where 991 patients previously treated for locally advanced or metastatic breast cancer with trastuzumab and a taxane were randomly assigned to receive either single-agent T-DM1 3.6 mg per kilogram of body weight intravenously 3-weekly or lapatinib plus capecitabine. The median progression-free survival (PFS) was 9.6 months with T-DM1 versus 6.4 months with the control regimen, and a hazard ratio for progression or death was 0.65 in favor of T-DM1 (95% CI 0.55 to 0.77). Importantly, patients assigned to T-DM1 lived longer (30.9 versus 25.1 months, respectively) and had fewer serious adverse events recorded. T-DM1 was associated with higher rates of thrombocytopenia and serum aminotransferase level elevations, whereas lapatinib and capecitabine were associated with more frequent diarrhea, nausea and palmar-plantar erythrodysesthesia [36]. These data led to approval of T-DM1 by the US Food and Drug Administration (FDA) in February 2013 for the treatment of patients with HER2-positive MBC who had previously received trastuzumab and a taxane.

In another randomized study (TDM4450g), where 137 patients with HER2-positive MBC or recurrent locally advanced breast cancer were assigned to either T-DM1 or trastuzumab plus docetaxel as first-line treatment, the median PFS was 14.2 months with T-DM1 and 9.2 months with trastuzumab plus docetaxel (hazard ratio 0.59; 95% CI 0.36 to 0.97) [37]. T-DM1 was associated with a more favorable safety profile with fewer serious adverse effects.

In the TH3RESA study, 602 patients with unresectable HER2-positive locally advanced breast cancer or MBC who had progressed on at least two prior HER2-directed regimens were randomly assigned to receive either T-DM1 or therapy chosen by the physician. Patients treated with T-DM1 achieved longer PFS (6.2 versus 3.3 months, respectively; hazard ratio 0.53, 95% CI 0.42 to 0.66) and longer survival (not reached versus 14.9 months), and had fewer severe (grade 3 or higher) adverse effects compared with a regimen chosen by the physician [38].

## Resistance to T-DM1

Despite these favorable efficacy results, most patients treated with T-DM1 eventually progress [36-38], and some HER2-positive breast cancers are primarily non-responsive or are only minimally responsive to T-DM1. Understanding of the resistance mechanisms is important for further development of T-DM1-directed therapies.

### T-DM1 resistance in preclinical models

Both primary and acquired resistance to T-DM1 have been observed in *in vitro* models of HER2-positive breast cancer and gastric cancer (Additional file 3) [17,39,40]. In *in vivo* preclinical models, efficacy of T-DM1 varied depending on the tumor mass in a trastuzumab- and lapatinib-resistant human breast cancer xenograft model (JIMT-1). While large (approximately 350 mm^3^) xenografts were resistant to T-DM1, small ones (approximately 70 mm^3^) were partially sensitive. T-DM1 inhibited remarkably well growth of very small JIMT-1 xenografts with no macroscopic tumor detected until resistance to T-DM1 emerged after prolonged treatment (16 weeks) with T-DM1 [18]. In another preclinical study, large HER2-positive human gastric xenografts (N-87) disappeared macroscopically totally with T-DM1, but microscopic deposits of residual tumor cells remained at the tumor inoculation sites. The residual cells had a low cell proliferation rate when stained for Ki-67, and survived T-DM1 treatment despite maintaining high HER2 protein expression [17]. These findings suggest that cancer relapse may occur after a long latency period despite macroscopically complete response to T-DM1.

### Primary and acquired resistance to T-DM1 in clinical trials

In a phase II study (TDM4558g) conducted in a cohort of 112 patients with HER2-positive MBC who had received prior chemotherapy and who had progressed on prior HER2-directed therapy or within 60 days after the last dose of trastuzumab, 29 (26%, 95% CI 18% to 34%) patients achieved objective response with single-agent T-DM1 (none had complete response) and 55 (49%) had stable disease [41]. In this study only 22 (20%) patients had disease progression as their best response, suggesting that most patients with HER2-positive MBC are not primarily resistant to T-DM1 despite prior exposure to HER2-directed therapy.

Primary resistance to T-DM1 may be more infrequent when the patients are naive to trastuzumab, although only indirect data are currently available to support this hypothesis. In the TDM4450g trial carried out in the first-line setting with most patients not previously treated with trastuzumab, 43 (64%, 95% CI 52% to 75%) out of the 67 patients with MBC treated with T-DM1 achieved objective response, including seven (10%) complete responders, and the median duration of response was not reached [37], whereas in the EMILIA trial conducted in the second-line setting in a patient population who had previously been treated with trastuzumab and a taxane, 169 (44%, 95% CI 39% to 49%) out of the 397 patients treated with T-DM1 had objective response, including four (1%) complete responders, and the median duration of response was 12.6 months [36].

While primary resistance to T-DM1 may be relatively infrequent, particularly in patients who have no prior exposure to trastuzumab, most initially responding patients eventually cease to respond despite continued treatment with T-DM1 [36-38], suggesting that acquired resistance to T-DM1 is a common problem.

### Potential factors that cause resistance to T-DM1

Except for low HER2 expression in cancer, the clinical, biological and pharmacological factors that are related to poor efficacy of T-DM1 are incompletely understood. Yet, factors that are strongly implicated in the biological mechanism of action of T-DM1 are good candidates for having a role in resistance to T-DM1.

**Figure 3:**
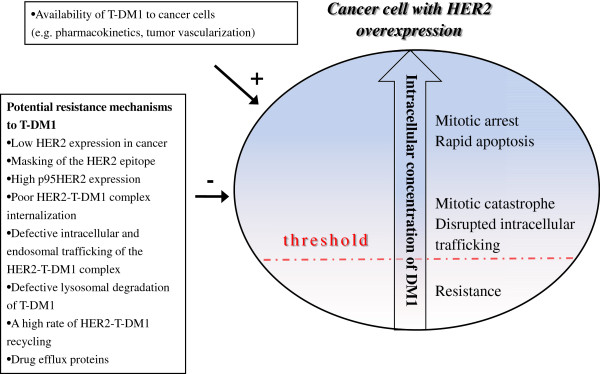
**Factors influencing the intracellular DM1 level.** DM1 may evoke cell death in a concentration-dependent manner, where a threshold concentration of intracellular DM1 and its metabolites needs to be exceeded for cell kill. At high DM1 concentrations mitotic arrest and rapid apoptotic death follow, whereas at lower levels mitotic catastrophe and disrupted intracellular trafficking occur, and at the lowest levels of DM1 cells show resistance. HER2, human epidermal growth factor receptor-2; T-DM1, trastuzumab emtansine.

DM1 and its metabolites (lysine-MCC-DM1) need to accumulate in cancer cells to reach a concentration that exceeds the threshold to evoke cell death [12]. Here we summarize the factors that may influence the intracellular DM1 concentration and thus cause resistance to T-DM1 (Figure 3, Table 2).

**Table 2:** Potential factors that may cause resistance to trastuzumab emtansine

**Factors decreasing intracellular DM1 level**	
T-DM1 binding to HER2	Low cancer HER2 expression
	HER2 down-regulation
	Shedding of HER2 ectodomain
	Masking of the trastuzumab binding epitope on HER2 p95HER2 expression
Intracellular trafficking and lysosomal degradation	Poor HER2-T-DM1complex internalization
	HER2-T-DM1 recycling to plasma membrane
	Failure of HER2 intracellular trafficking
	Inefficient lysosomal degradation of T-DM1
Drug efflux	MDR1 expression
**Other factors**	
Altered DM1 target	Beta1-tubulin mutation
Autocrine or stromal growth factors	Overexpression of a beta3-tubulin isoform
Modulators of the apoptotic pathway	Microtubule-associated proteins
Activation of cell survival pathways	

HER2, human epidermal growth factor receptor-2; T-DM1, trastuzumab emtansine.

#### Low tumor HER2 expression

Expression of HER2 on cancer cells is essential for T-DM1 efficacy. Not surprisingly, retrospective analyses of two phase II trials (TDM4258g and TDM4374g) carried out in advanced breast cancer revealed that patients with HER2-positive cancer (defined either as immunohistochemistry (IHC) 3+ or fluorescence *in situ* hybridization +) had more frequent responses to T-DM1 than patients who had HER2-normal cancer; in TDM4258g the objective response rates were 34% and 5%, respectively, and in TDM4374g, 41% and 20%, respectively [41-43]. When cancer HER2 mRNA levels were quantified by quantitative reverse transcriptase polymerase chain reaction in the subgroup of HER2 IHC 3+ disease, patients with the median or higher HER2 mRNA concentration responded more often to T-DM1 than those with a lower concentration (in TDM4374g, the response rates were 50% and 33%, and in TDM4258g, 36% and 28%, respectively) [41-43]. Quantitative HER2 assays should probably be performed from the most recent cancer biopsy tissue material rather than the primary tumor, since the primary tumor HER2 content may sometimes be discordant with that of most metastatic lesions [44,45].

#### Poor internalization of the HER2-T-DM1complexes

Binding of T-DM1 to the extracellular domain of HER2 triggers entry of the HER2-T-DM1 complex into cancer cells via receptor-mediated endocytosis [12,13]. A high rate of complex internalization may result in high intracellular concentrations of DM1, and deceleration of the endocytosis rate might cause loss of sensitivity to T-DM1. However, it is unknown whether the rate of internalization differs between cancers, and the factors affecting the rate have not been identified.

#### Defective intracellular and endosomal trafficking of the HER2-T-DM1 complex

The internalized endocytotic vesicles containing HER2-T-DM1 complexes fuse and form early endosomes. The contents of early endosomes can be recycled back to the cell membrane, or the early endosome can mature into a lysosome [13] where proteolytic degradation of the antibody part of T-DM1 occurs (Figure 1). The dynamics of loading of the lysosomes with the HER2-T-DM1 cargo may influence the intracellular DM1 levels. T-DM1 treatment results in defective intracellular trafficking of the HER2 protein [18], which is not in disagreement with a hypothesis that mitosis is not the only target of anti-microtubule agents, but rather trafficking on the microtubules [34].

#### Defective lysosomal degradation of T-DM1

Since DM1 release in the cytosol occurs only following proteolytic degradation of the trastuzumab part of the T-DM1 complex in the lysosomes, efficient lysosomal degradation is essential. Expression and activity of lysosomal enzymes may vary between tumors and even cancer cells, and is influenced by several factors such as tumor necrosis factor-α, lysosomal vacuolar H^+^-ATPase (V-ATPase), and Bax inhibitor-1 [46-48]. All of these factors may thus affect cancer sensitivity to T-DM1. For example, V-ATPase inhibition using archazolid, an inhibitor of myxobacterial origin, results in apoptosis, growth inhibition, and impaired HER2 signaling in the trastuzumab-resistant cell line JIMT-1 [49].

#### Masking of the HER2 epitope

The trastuzumab binding epitope of HER2 can be masked at least partly by MUC4 or hyaluronan inhibiting the binding of trastuzumab to HER2 [20,21]. Although no similar data are available regarding T-DM1, masking of the epitope may also decrease the binding of T-DM1 to HER2.

#### High p95HER2 expression

p95HER2 is an amino-terminally truncated form of HER2 that lacks most of the extracellular domain of the protein, including subdomain IV recognized by trastuzumab. Therefore, trastuzumab or T-DM1 cannot bind to p95HER2 [23]. No studies have thus far correlated breast cancer p95HER2 expression with sensitivity to T-DM1.

#### A high rate of HER2-T-DM1 recycling

After internalization, trastuzumab-HER2 complexes can evade degradation and undergo rapid and efficient recycling to the cell membrane. About 50% of internalized HER2-bound trastuzumab is recycled back to the cell membrane within 5 minutes and 85% within 30 minutes in *in vitro* breast cancer cell culture [50]. It is currently unknown whether cytoplasmic recycling of T-DM1 differs from that of trastuzumab. Extensive recycling of T-DM1 could yet lead to decreased efficacy, since in the absence of proteolytic degradation of trastuzumab no release of intracellular DM1 can occur.

#### Drug efflux pumps

MDR1 (also known as P-glycoprotein) is an ATP-dependent transporter that mediates efflux of drugs and toxins from the cell. Tumor MDR1 expression is associated with poor response to chemotherapy in many types of cancer [51,52]. DM1 and other maytansinoids are substrates of MDR1, and MDR1 expression is linked with a maytansine-resistant cancer phenotype [53]. In one study, one out of three T-DM1-resistant breast cancer cell lines showed upregulation of multi-drug resistance transporters [40], but the role of drug efflux proteins in resistance to T-DM1 may be complex and requires further study [39].

#### Neuregulin-HER3 signaling

Presence of the HER3 ligand neuregulin-1β (NRG-1β, heregulin) suppressed the cytotoxic activity of T-DM1 in four out of the six breast cancer cell lines tested, this effect being reversed by pertuzumab [54]. Activating *PIK3CA* mutations were present in the two breast cancer cell lines where NRG-1β did not inhibit T-DM1 activity, while the four cell lines where T-DM1 activity was reduced did not harbor *PIK3CA* mutations [54]. As trastuzumab, T-DM1 suppresses the phosphatidylinositol 3′-kinase (PI3K) signaling pathway [40]. The potential association between *PIK3CA* mutational status and T-DM1 efficacy remains unknown, but the results from clinical breast cancer series suggest that trastuzumab benefit does not depend on the mutational status of *PIK3CA*[55,56] or tumor PTEN expression [57].

#### Altered tubulins

Since DM1 binds to tubulin, altered or mutant tubulins [58,59] or altered modulators of the microtubule dynamics might also impact on the response to T-DM1 [39,47].

### Concentration-dependent mechanism of action of free intracellular DM1

A high intracellular concentration of DNA damaging agents often leads to terminal mitotic arrest and apoptosis [60,61]. Besides apoptosis, aberrant cytokinesis (pathological mitoses) and multinucleation may take place at low concentrations of DNA damaging agents [60-62], which is called mitotic catastrophe [60,63].

T-DM1 caused rapid tumor shrinkage of human gastric cancer xenografts with high HER2 expression (IHC 3+), the type of cell death being predominantly apoptosis [17], whereas T-DM1 was less effective on human breast cancer xenografts expressing moderate HER2 levels (IHC 2+), but prolonged treatment times eventually evoked apoptosis and mitotic catastrophe in these xenografts [18]. T-DM1 may thus cause cell death through two molecular mechanisms depending on the intracellular DM1 concentration, high concentrations of DM1 causing mitotic arrest with no or few mitotic catastrophes followed by apoptosis, whereas cell exposure to low DM1 concentrations of long duration may lead to mitotic catastrophes and cell death. Prolonged T-DM1 treatment led to disruption of intracellular trafficking of HER2 in the breast cancer xenografts with moderate HER2 expression (IHC 2+) [18].

Based on these findings, we hypothesize that the anti-cancer effects of T-DM1 depend on the intracellular concentration of DM1 and the duration of exposure. When the intracellular concentration of DM1 exceeds a critical threshold level, mitotic arrest and rapid apoptotic death follows, whereas mitotic catastrophe and disrupted intracellular trafficking occur at lower DM1 levels provided that the exposure time is long enough (Figure 3). This hypothesis requires further research in preclinical models, but it could support carrying out clinical trials evaluating prolonged administration of T-DM1 in cancer patient populations with low to moderate tumor HER2 expression levels.

## Strategies to improve T-DM1 efficacy and circumvent resistance

Here we summarize the potential strategies to improve efficacy of T-DM1 and to prevent drug resistance. Some of these strategies are already being tested in clinical trials.

### T-DM1 in the adjuvant and neoadjuvant setting

At present T-DM1 has been approved by the FDA for second-line treatment of HER2-positive MBC. Ongoing clinical trials are evaluating the potential role of T-DM1 as first-line treatment of MBC and in the adjuvant and neoadjuvant settings [64]. The trials to be carried out in patient populations with a small or minimal tumor bulk are clearly of great importance, since T-DM1 has substantial efficacy and a favorable safety profile as a single agent in advanced breast cancer, and T-DM1 may be particularly effective in eradication of cancer when the tumor mass is small [65].

### Combination therapies with T-DM1

There is substantial interest in investigating the efficacy and safety of T-DM1 in combination with other anti-cancer agents, particularly with those that have proved effective in combination with trastuzumab. Both paclitaxel and docetaxel are approved for the treatment of HER2-positive MBC in combination with trastuzumab [4,66]. Since DM1 and taxanes bind to tubulins at different sites [12,67], a combination of taxanes and T-DM1 could have synergistic effects. Two ongoing clinical trials are evaluating such combinations (NCT00951665 and NCT00934856).

An ongoing clinical trial (NCT01702558) evaluates efficacy and safety of capecitabine plus T-DM1 in MBC. This trial is built on the clinical activity observed in a phase II single cohort study that evaluated the combination of capecitabine and trastuzumab in HER2-positive MBC [68], and a randomized phase II trial that compared a combination of capecitabine, trastuzumab and docetaxel to trastuzumab plus docetaxel, the triple combination resulting in significantly improved PFS [69].

Patients with HER2-positive MBC treated with pertuzumab in combination with trastuzumab and docetaxel had longer PFS and overall survival compared with patients who received placebo, trastuzumab and docetaxel in a large randomized trial (CLEOPATRA) [70]. The ongoing trials evaluating the combinations of pertuzumab plus T-DM1 and the triple combination of pertuzumab plus T-DM1 plus a taxane are thus well founded [64]. MARIANNE (NCT01120184) is an ongoing trial with a planned target population of over 1,000 patients with HER2-positive MBC. In this study, patients who have not received prior chemotherapy for MBC are randomly assigned to receive T-DM1 plus placebo, T-DM1 plus pertuzumab, or trastuzumab plus paclitaxel or docetaxel. The combination of T-DM1 and lapatinib also deserves clinical evaluation considering the superior efficacy of lapatinib and trastuzumab in HER2-positive MBC over lapatinib alone [71].

Trastuzumab has been approved for the treatment of patients with HER2-positive and hormone receptor-positive postmenopausal MBC in combination with an aromatase inhibitor [72,73]. The efficacy and safety of T-DM1 is being investigated in combination with endocrine therapy (with tamoxifen in premenopausal women and aromatase inhibitor in postmenopausal women) as neoadjuvant treatment of HER2-positive and hormone receptor-positive operable breast cancer (NCT01745965).

GDC-0941, a selective and potent PI3K inhibitor, was effective in preclinical models of trastuzumab-resistant breast cancer, where administration of GDC-0941 in combination with HER2-directed treatment (trastuzumab, pertuzumab, or lapatinib) inhibited in a synergistic fashion growth of breast cancer cells [74,75]. In an ongoing dose escalation phase Ib study (NCT00928330), the safety, tolerability, pharmacokinetics, and efficacy of T-DM1 and GDC-0941 are being investigated in patients with HER2-positive MBC who have progressed on prior trastuzumab therapy.

### Circumventing MDR1-mediated resistance by a modified linker

Since the maytansinoids are substrates for the MDR1 transporters [53], drug efflux by MDR1 may decrease the intracellular DM1 concentration, resulting in a decline in efficacy. Kovtun and colleagues [53] developed a potential strategy to circumvent MDR1-mediated resistance to T-DM1 by attaching DM1 to an antibody using a hydrophilic linker, PEG_4_Mal. The degradation of such conjugates in cancer cells resulted in the release of lysine-PEG_4_Mal-DM1 instead of lysine-MCC-DM1 (the active metabolite of T-DM1). Lysine-PEG_4_Mal-DM1 is a poor substrate of MDR1, and the conjugates with the PEG_4_Mal linker evaded MDR1-mediated resistance both in MDR1-expressing cells *in vitro* and in MDR1-expressing xenografts *in vivo*[53]. Therefore, MDR1 drug transporter-mediated resistance to T-DM1 might be overcome by replacing the SMCC linker with the PEG_4_Mal linker.

### Modulation of HER2 recycling

When intracellular HER2 is recycled to the plasma membrane, trastuzumab recycles as a part of the HER2-trastuzumab complex [50]. Heat shock protein (Hsp)90 is a molecular chaperone that participates in the regulation of HER2 recycling. Geldanamycin, an inhibitor of Hsp90, reduces HER2 recycling and results in an over three-fold increase in the concentration of the HER2-trastuzumab complex being retained in tumor cells [50]. Geldanamycin redistributes cell surface HER2 into the internal vesicles of the endosomes, enhancing proteolytic degradation of HER2 [50,76].

It has currently not been established whether intracellular T-DM1 is also recycled, but inhibition of recycling is of potential interest from the therapeutic point of view. Hypothetically, sequential administration of T-DM1 followed by geldanamycin (or one of its derivatives [77]) might inhibit recycling of T-DM1 and direct the HER2-T-DM1 complexes for lysosomal degradation, thus increasing the intracellular levels of DM1 and cytotoxicity. Sequential administration of the two drugs in this order could be important, since Hsp90 inhibitors decrease cell surface HER2 and might reduce T-DM1 binding to cells [50].

### Modification of the cytotoxic drug moiety

Since the intracellular DM1 concentration is crucial for the cell-killing potency of T-DM1, delivery of greater quantities of DM1 into the tumor cells would likely improve efficacy. This could be achieved using more heavily loaded T-DM1 that delivers more cytotoxic drug to the target cells. However, increasing the drug-antibody ratio (DAR) of an ADC usually results in a faster clearance of the ADC. ADCs with a DAR of 2 to 4 have a more favorable pharmacokinetic profile than those with a higher DAR [78,79]. Therefore, increasing the number of DM1 molecules from the average of 3.5 per one trastuzumab might result in a shorter half-life and destabilization of the complex, and decreased efficacy. Alternative strategies include binding of another cytotoxic drug in addition to DM1 to trastuzumab, or administering another ADC in combination with T-DM1, such as a cytotoxic drug linked to pertuzumab. The second cytotoxic drug could have an alternative (non-tubulin) mechanism of action [39].

### Improving the Fc part of trastuzumab

Trastuzumab retains its anti-cancer activity when conjugated to DM1, and improving the antibody component of the conjugate could thus result in a more efficient ADC. Afucosylated trastuzumab has superior efficacy compared with trastuzumab in some preclinical breast cancer models [80], and amino acid modifications of the Fc part of trastuzumab may also improve efficacy [81]. Yet, DM1 remains a key component regarding the overall anti-tumor activity of T-DM1.

### Radioimmunotherapy conjugates

Auger electron emitting ^111^In-NLS-trastuzumab is effective in the treatment of trastuzumab-resistant breast cancer cells [82]. Radioimmunotherapy conjugates might find a role in the treatment of patients who have failed T-DM1 therapy.

## Conclusion

T-DM1 is a valuable new agent for the treatment of HER2-positive breast cancer. T-DM1 has several mechanisms of action consisting of the anti-tumor effects associated with its key components, trastuzumab and the cytotoxic drug DM1. Clinical research carried out suggests superior efficacy of T-DM1 compared with trastuzumab or trastuzumab plus chemotherapy in the treatment of MBC. However, both primary and secondary resistance to T-DM1 occurs. In addition to the identified resistance mechanisms related to trastuzumab, several factors that influence the intracellular DM1 concentration may confer resistance to T-DM1. Understanding of these factors may lead to the development of strategies that improve efficacy of T-DM1 and may circumvent drug resistance.

## Note

This article is part of a series on *‘Recent advances in breast cancer treatment*’*,* edited by Jenny Chang. Other articles in this series can be found at http://breast-cancer-research.com/series/treatment.

## Abbreviations

ADC: Antibody-drug conjugate; DAR: Drug-antibody ratio; DM1: Derivative of maytansine 1; FDA: Food and drug administration; HER2: Human epidermal growth factor receptor-2; Hsp: Heat shock protein; IHC: Immunohistochemistry; MBC: Metastatic breast cancer; PFS: Progression-free survival; PI3K: Phosphatidylinositol 3′-kinase; SMCC: *N*-succinimidyl-4-(*N*-maleimidomethyl)cyclohexane-1-carboxylate; T-DM1: Trastuzumab-emtansine; V-ATPase: Vacuolar H^+^-ATPase.

## Competing interests

The authors declare that they have no competing interests.

## Supplementary Material

Additional file 1 **Time-lapse microscopy of SKBR-3 breast cancer cells grown with 1 μg/mL trastuzumab or T-DM1.** SKBR-3 is a HER2-positive, trastuzumab-sensitive breast cancer cell line. SKBR-3 is much more sensitive to T-DM1 than to trastuzumab. Images were taken by Cell-IQ® system (Chip-Man Technologies Ltd, Tampere, Finland). Click here for file

Additional file 2 **Time-lapse microscopy of JIMT-1 breast cancer cells grown with 1 μg/mL trastuzumab or T-DM1.** JIMT-1 is a HER2-positive, trastuzumab and lapatinib cross-resistant breast cancer cell line. JIMT-1 is sensitive to T-DM1. Images were taken by Cell-IQ® system (Chip-Man Technologies Ltd, Tampere, Finland). Click here for file

Additional file 3 **Time-lapse microscopy of SNU-216 gastric cancer cells grown with 1 μg/mL trastuzumab or T-DM1.** SNU-216 is a HER2-positive, trastuzumab-resistant gastric cancer cell line. SNU-216 is resistant to trastuzumab and T-DM1. Images were taken by Cell-IQ® system (Chip-Man Technologies Ltd, Tampere, Finland). Click here for file
